# Successful isolation of persistent left superior vena cava using very high-power, short-duration radiofrequency ablation: Case report

**DOI:** 10.1016/j.hrcr.2025.10.013

**Published:** 2025-10-17

**Authors:** Takeshi Mori, Kazuo Kato, Hiroko Goto, Shinjiro Miyata, Nobuo Ishiguro, Makito Kaneshiro

**Affiliations:** Department of Cardiology, Nagoya Tokushukai General Hospital, Kasugai, Aichi, Japan

**Keywords:** Persistent left superior vena cava, Atrial fibrillation, Very high-power, short-duration, High-frequency stimulation, Left atrial appendage, Case report

**Key Teaching Points**

▪ Persistent left superior vena cava (PLSVC) is a relatively common venous anomaly that may serve as a nonpulmonary vein trigger in atrial fibrillation (AF) and, when identified, may warrant electrical isolation to prevent AF recurrence.
▪ Very high-power, short-duration (VHPSD) ablation offers a promising strategy for PLSVC isolation owing to its ability to create shallow and wide lesions while minimizing the risk of damaging adjacent structures such as the phrenic nerve and left atrial appendage (LAA).
▪ Here, PLSVC isolation was successfully achieved using VHPSD ablation, with preablation pacing confirming the absence of phrenic nerve capture, and no procedural complications were observed.
▪ The anatomical proximity between the PLSVC and the LAA poses a risk of inadvertent LAA isolation or dysfunction, particularly with other energy modalities such as pulsed-field ablation; VHPSD ablation may mitigate these risks.
▪ This report is, to our knowledge, the first to describe the safe use of VHPSD ablation for PLSVC isolation, and it highlights the need for further studies to evaluate its long-term efficacy and safety in broader clinical populations.

## Introduction

Persistent left superior vena cava (PLSVC) is a relatively common thoracic venous anomaly, with an estimated prevalence of approximately 0.2%–0.6%.^1^ It is identified in approximately 0.9% of patients undergoing catheter ablation for atrial fibrillation (AF).^2^ PLSVC can be a nonpulmonary vein focus for AF initiation.^3^ Regardless of its demonstrable arrhythmogenicity, empirical isolation of the PLSVC is considered useful for preventing recurrence of AF.^4^ However, because of the relatively low prevalence of PLSVC, a standardized method for its isolation has yet to be established.^2^ Very high-power, short-duration (VHPSD) ablation produces shallow and wide lesions.^5^ Given the anatomical proximity of the phrenic nerve and the left atrial appendage (LAA) to the PLSVC, VHPSD ablation may be a viable approach for PLSVC isolation. Although PLSVC isolation using cryoballoon ablation and pulsed-field ablation (PFA) has been reported,^6^^,^^7^ no reports have described the use of VHPSD ablation for this purpose. To our knowledge, we present the first documented case of persistent AF with the PLSVC, wherein PLSVC isolation was successfully performed using VHPSD ablation without complications.

## Case report

An 86-year-old man presented to our hospital with shortness of breath as his chief complaint. A 12-lead electrocardiogram revealed AF, leading to the diagnosis of symptomatic persistent AF. Despite his advanced age, the patient had been independently performing his activities of daily living, had a Clinical Frailty Scale score of approximately 2, and expressed a desire for curative treatment via catheter ablation. Transthoracic echocardiography revealed an enlarged coronary sinus, whereas preprocedural contrast-enhanced computed tomography identified a PLSVC without communication with the right superior vena cava (RSVC) or left pulmonary vein.

**Figure 1:**
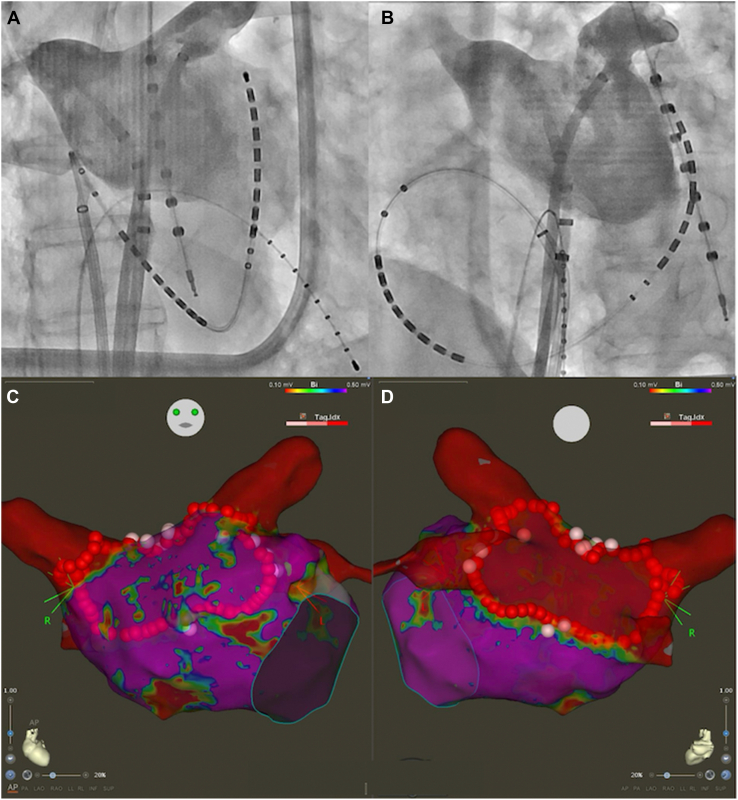
Left atrial angiography and box-shaped pulmonary vein isolation. (**A**) Right and (**B**) left anterior oblique views. Box-shaped pulmonary vein isolation was performed as shown in the (**C**) anterior and (**D**) posterior views.

**Figure 2:**
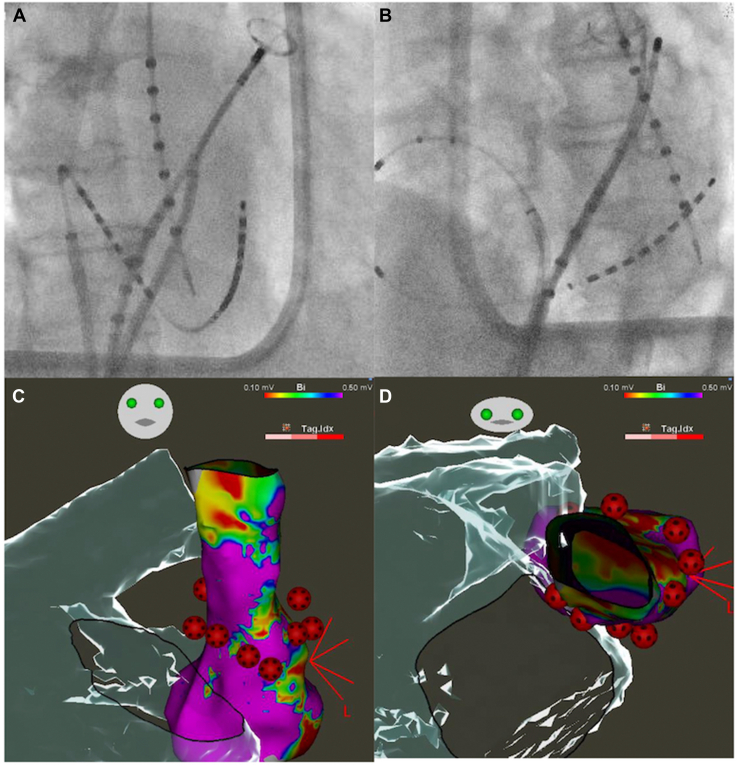
Persistent left superior vena cava (PLSVC) mapping and ablation points. The LASSO catheter was positioned superior to the left atrial roof to monitor electrical potentials during the procedure, as shown in panels (**A**) and (**B**). After high-density mapping with the OCTARAY catheter, radiofrequency energy was delivered to the PLSVC using a very high-power, short-duration strategy. Before ablation, high-output pacing at 10 V × 5 ms was performed to assess phrenic nerve capture; however, no diaphragmatic stimulation was observed around the entire circumference. (**A**) Right and (**B**) left anterior oblique views, and (**C**) anterior and (**D**) cranial views.

**Figure 3:**
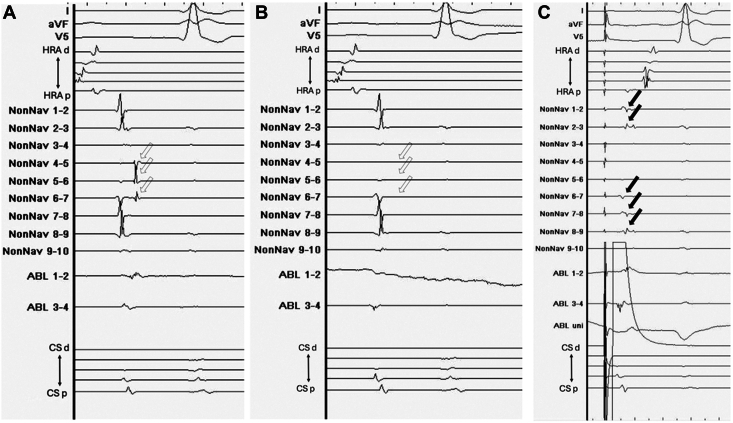
Intracardiac electrogram changes in the persistent left superior vena cava (PLSVC) during radiofrequency energy delivery. (**A**) Preisolation potential recorded on the LASSO catheter within the PLSVC. (**B**) Postisolation potential recorded on the LASSO catheter within the PLSVC. (**C**) Potential recorded on the LASSO catheter during pacing from the distal site of the coronary sinus catheter. Circumferential ablation within the PLSVC was performed using a very high-power, short-duration strategy, which resulted in the elimination of the potentials indicated by the *white arrow* in panel A, as shown in panel B. Furthermore, as shown in panel C, pacing from the distal site of the coronary sinus catheter did not delay the residual potentials recorded on the LASSO catheter (*black arrow*), supporting the interpretation that these signals represent far-field potentials from the left atrial appendage.

**Figure 4:**
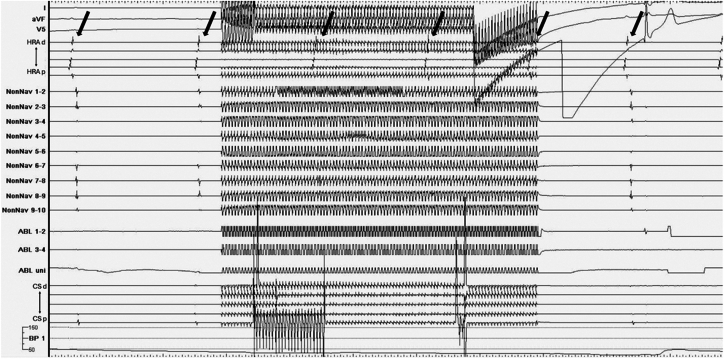
Dormant exit block revealed by high-frequency stimulation in the persistent left superior vena cava (PLSVC). The dormant exit block was revealed by high-frequency stimulation (20 Hz, 20 V × 10 ms) after adenosine triphosphate administration at the 9-o’clock position of the PLSVC. As indicated by the *black arrows*, atrial electrograms were observed at regular intervals during sinus rhythm, suggesting that burst pacing within the PLSVC did not occur in the atrium. This indicates the formation of a more durable exit block at this site.

**Figure 5:**
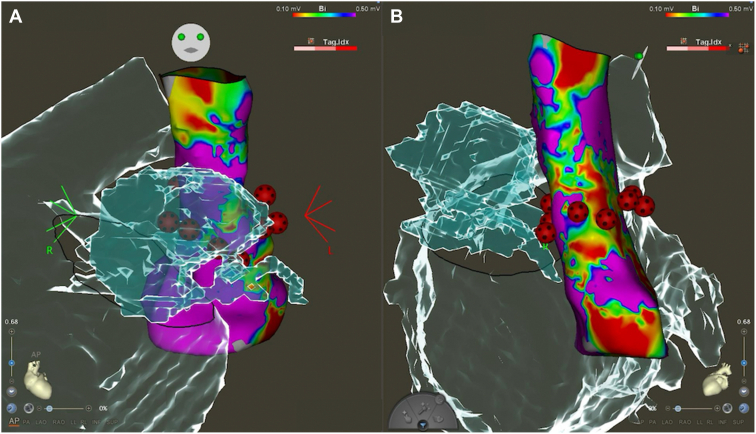
Anatomical relationship between the ablation sites and the left atrial appendage (LAA). The persistent left superior vena cava (PLSVC) and LAA are anatomically close. Therefore, potential effects on the LAA should be carefully considered during ablation within the PLSVC.

**Supplemental Figure 1:**
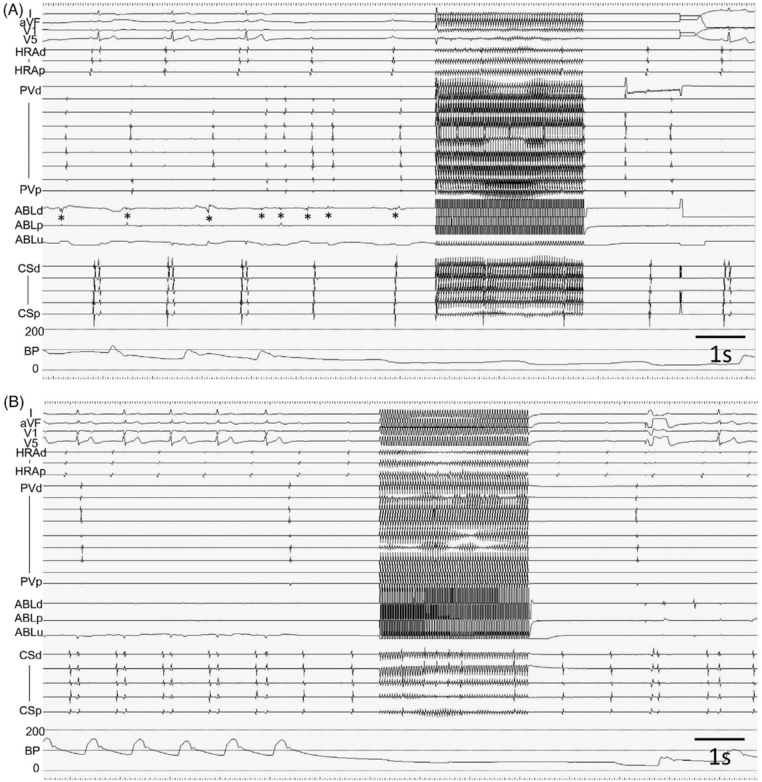
Intracardiac recordings were obtained during HFS on the LAPW in SR while inducing an atrioventricular block through ATP administration following Box-PVI in cases with both complete (A) and incomplete Box-PVI (B). A: The atrial rhythm remained unchanged by the HFS, indicating an HFS (-) response. B: The atrial rhythm was influenced by the HFS, showing an HFS (+) response, which suggests dormant "exit" conduction, although non-conducted automatic firing was noted before and after the HFS.

Catheter ablation was performed 10 days after the presentation of the patient to our hospital. A CARTO3 mapping system and a CARTOSOUND module (Biosense Webster, Diamond Bar, CA) were used to reconstruct the geometry of the left atrium with an OCTARAY catheter (Biosense Webster). Box-shaped pulmonary vein isolation was performed using a QDOT MICRO contact force–sensing catheter (Biosense Webster) (Figure 1). The entrance block of both pulmonary veins and the posterior wall of the left atrium was confirmed using the OCTARAY catheter, and the exit block was verified using high-frequency stimulation (HFS), with pacing at 20 Hz and 20 V × 10 ms. HFS enables confirmation of the exit block by demonstrating the absence of conduction in both the endocardial and epicardial sides,^8^^,^^9^ thereby providing more robust evidence of the exit block.^10^ Empirical cavotricuspid isthmus ablation was performed, and a bidirectional block was confirmed. No myocardial sleeves were identified in the RSVC using the OCTARAY catheter. Mapping of the PLSVC was conducted under sinus rhythm using the OCTARAY catheter, which revealed a long sleeve in the PLSVC on the roof of the left atrium (Figure 2). Considering conduction to the left atrium, we opted for isolation at the distal segment of the PLSVC rather than at the mid-portion. We placed a LASSO catheter (Biosense Webster) at the distal PLSVC around the left atrial roof to assess the nearby electrical potential. The dorsal aspect was defined as the 12-o’clock position in the superior view. Before ablation, pacing was performed at 20 V × 1 ms to confirm the absence of phrenic nerve capture. Radiofrequency energy was delivered using the VHPSD protocol (90 W for 4 seconds) with a contact force of approximately 5–15 g (Figure 2). As phrenic nerve capture was not observed in any direction of the PLSVC, radiofrequency energy was delivered circumferentially using the VHPSD strategy. This approach eliminated the latter potentials recorded on the LASSO catheter (Figure 3A and 3B). In addition, pacing from the distal site of the coronary sinus catheter did not delay the LASSO potentials, indicating that the residual signals likely represented far-field potentials originating from the LAA (Figure 3C). The exit block was confirmed by HFS at all sites except the 7-o’clock position, and a dormant exit block was confirmed using HFS at the 9-o’clock position (Figure 4) (Supplemental Figure 1).^10^ Although the exit block could not be demonstrated at the 7-o’clock position, no local electrograms were recorded within the PLSVC, suggesting far-field capture from the adjacent LAA. Fusion of preprocedural contrast-enhanced computed tomography images confirmed that the PLSVC and LAA were anatomically close (Figure 5). After intravenous bolus administration of 10 μg of isoproterenol, atrial burst pacing was performed, with the pacing cycle length reduced to 170 ms, confirming noninducibility of any arrhythmias. Accordingly, no additional intervention targeting the PLSVC was undertaken to avoid energy application to the mid- and proximal portions.

Postoperative transthoracic echocardiography revealed no pericardial effusion, with no signs of cerebral infarction or phrenic nerve palsy. The patient was discharged on postoperative day 3. Although early recurrence of AF was observed at the first outpatient visit after discharge on day 14, treatment with bepridil at 100 mg/d was initiated. At an outpatient visit 2 weeks later, the patient was found to have converted to sinus rhythm. Subsequent follow-ups in the outpatient clinic over 6 months revealed no recurrence of AF, even after discontinuing bepridil, except for β-blockers.

## Discussion

The phrenic nerve runs anterolateral to the PLSVC^11^; therefore, caution is warranted to avoid phrenic nerve palsy when isolating PLSVC mid-to-distal segments. Although the frequency is low, cases of phrenic nerve palsy during PLSVC isolation have been reported.^3^^,^^12^ Phrenic nerve palsy is a high-risk complication associated with PLSVC isolation, particularly when cryoballoon ablation is used.^13^ In this case, we carefully confirmed the absence of phrenic nerve capture by pacing the ablation catheter before each energy delivery, and no phrenic nerve palsy was observed. Because the phrenic nerve was not captured, it is unlikely that it is involved in the lesion by energy delivery. Nonetheless, given that VHPSD ablation creates shallower lesions,^5^ it is theoretically less likely to cause phrenic nerve damage, suggesting its utility for PLSVC isolation.

PFA is known for its tissue specificity; however, this approach raises concerns regarding potential complications such as coronary artery spasm.^14^ Laryngospasms have also been reported during PFA targeting the left superior pulmonary vein.^15^ Furthermore, PFA targeting the right superior pulmonary vein has reportedly been associated with the emergence of low-voltage areas in the superior vena cava.^16^ Considering the anatomical relationship between the PLSVC and the LAA (Figure 5) and the difficulty in predicting the depth of lesion formation with PFA (similar to that in cryoballoon ablation), PFA delivered from the PLSVC may inadvertently result in isolation of the LAA or impair its function. Because VHPSD ablation creates a shallower and wider lesion compared with conventional ablation,^5^ it may have a less significant impact on the adjacent LAA. In addition, a shorter ablation time can reduce the overall procedure time. Therefore, VHPSD ablation is a rational treatment method for PLSVC isolation.

Because empirical isolation of the PLSVC has been reported to be effective in preventing AF recurrence,^4^ distal PLSVC empirical isolation was performed here. Proximal or mid-portion isolation requires extensive energy application and is technically challenging.^12^^,^^17^ As the patient was an older adult and minimizing the procedure time was a priority, distal PLSVC isolation was performed first. Subsequently, isoproterenol was used to induce AF; nevertheless, as AF was not provoked, isolation at the proximal or mid-portion was not performed. As displayed in Figure 2, gaps were observed between the ablation points in the PLSVC. This occurs because VHPSD ablation forms a wide lesion,^6^ making it unnecessary to ablate the entire circumference, even when isolating the RSVC, as performed at our hospital. Therefore, VHPSD ablation is considered to reduce the procedure time.

As isolation in our case was limited to the distal PLSVC, the efficacy of VHPSD ablation at the mid-PLSVC could not be evaluated. In certain cases, isolation of the mid-PLSVC may be necessary; however, further studies are warranted to determine the efficacy of VHPSD ablation in such scenarios. This study specifically reports on the efficacy of VHPSD ablation at the distal PLSVC.

## Conclusion

We report a case of successful PLSVC isolation using VHPSD ablation. Considering the need to avoid complications such as phrenic nerve palsy, inadvertent isolation of the LAA, and impaired LAA function, VHPSD ablation appears to be an effective modality for PLSVC isolation. Further large-scale studies are warranted to evaluate the efficacy and safety of PLSVC isolation with each modality.
